# Correction: A diel multi-tissue genome-scale metabolic model of *Vitis vinifera*

**DOI:** 10.1371/journal.pcbi.1014135

**Published:** 2026-03-27

**Authors:** Marta Sampaio, Miguel Rocha, Oscar Dias

There are errors in the Funding statement. The correct Funding statement is as follows: Portuguese Foundation for Science and Technology (FCT) supported this work under the scope of the strategic funding of UID/BIO/04469/2020 unit and the PhD scholarship (SFRH/BD/144643/2019) to M.S. FCT also celebrated an Assistant Research contract with O.D. obtained under CEEC Individual 2018 (DOI 10.54499/CEECIND/03425/2018/CP1581/CT0020). This study was also supported by LABBELS - Associate Laboratory in Biotechnology, Bioengineering and Microelectromechanical Systems, LA/P/0029/2020. The funders had no role in study design, data collection and analysis, decision to publish, or preparation of the manuscript.
