# Supplementary material for: A diel multi-tissue genome-scale metabolic model of Vitis vinifera
Source: PLoS Comput Biol. 2024 Oct 10;20(10):e1012506. doi: 10.1371/journal.pcbi.1012506 (PMC11495577; doi:10.1371/journal.pcbi.1012506)
Supplement: S1 File — (PDF) [file pcbi.1012506.s001.pdf]

Supplementary Material

A diel multi-tissue genome-scale metabolic model of *Vitis vinifera*

Marta Sampaio[1,], Miguel Rocha[1,2], and Oscar Dias[1,2]

[1]Centre of Biological Engineering, University of Minho, Campus of Gualtar, 4710-057, Braga, Portugal

[2]LABBELS, Associate Laboratory, Braga/Guimarães, Portugal

Contents

Supplementary Material 1

1. iplants repository 2
2. Model reconstruction 4
3. Model properties 5
   3.1. Unique reactions 5
   3.2. Specialized metabolic pathways 5
4. Tissue-specific models 9
   4.1. Differential flux analysis 9
5. Diel multi-tissues: 11
   5.1. Sulfate assimilation 11
   5.2. Nitrate assimilation 13
6. Machine Learning and Fluxomics 17
   6.1. Unsupervised analysis 17
   6.2. Supervised analysis 18

References 21

List of Figures

Fig. S1. Neo4j database schema representing the relationships between the data 3

Fig. S2. The main steps of the *V. vinifera* GSMM reconstruction 4

Fig. S3. Pathway distribution of reactions included in the *V. vinifera* model and not in the other plant models 5

Fig. S4. Simplified schema of the phenylpropanoid pathway in the *V. vinifera* model................ 6
Fig. S5. Simplified schema of the terpenoid biosynthesis pathway in the V. vinifera model........ 7
Fig. S6. Simplified schema of the main metabolic pathways in the model affected by varying nitrate levels.. 16
Fig. S7. t-SNE visualization of the fluxomics data obtained from the 73 context-specific GSMMs 17
Fig. S8. Beeswarm plot of SHAP values for the reactions that contribute most to KNN's predictions 18

List of Tables

Table S1. Fluxes for the metabolites stored between light and dark phases in the diel multi-tissue model with mature berry 11

1. iplants repository

To collect and organize all the relevant data for the model reconstruction efforts, a repository with the metabolic information of PlantCyc and MetaCyc databases and UniProt sequence data was created. In total, the repository includes 24333 metabolites, 205128 reactions, 3519 pathways, and 22433 enzymes, 72% of which have a protein sequence.

The repository includes two database management systems: the Neo4j database saves the relationships between metabolic entities, while MongoDB saves all the metadata that characterises the entities. The schema of the Neo4j database is shown in Fig. S1.

Besides metabolites, reactions, pathways, and enzymes, it includes nodes for genes, organisms, and metabolic models. Regarding relationships, the reactions are linked to metabolites by two different types of edges that characterise and distinguish their reactants and products. Pathway and enzyme nodes are also linked to reactions and organisms. An enzyme can be a protein complex and be composed of several monomers; thus, enzyme nodes can be linked. In addition, genes are linked to enzymes and an

organism node. Finally, metabolic models are linked to an organism and to metabolites, reactions, and enzymes that define the model content.

The MongoDB database includes collections for the different nodes of Neo4j. The considered data attributes differ, depending on the collection. Details on how these data are organized are available in Supplementary File 1.

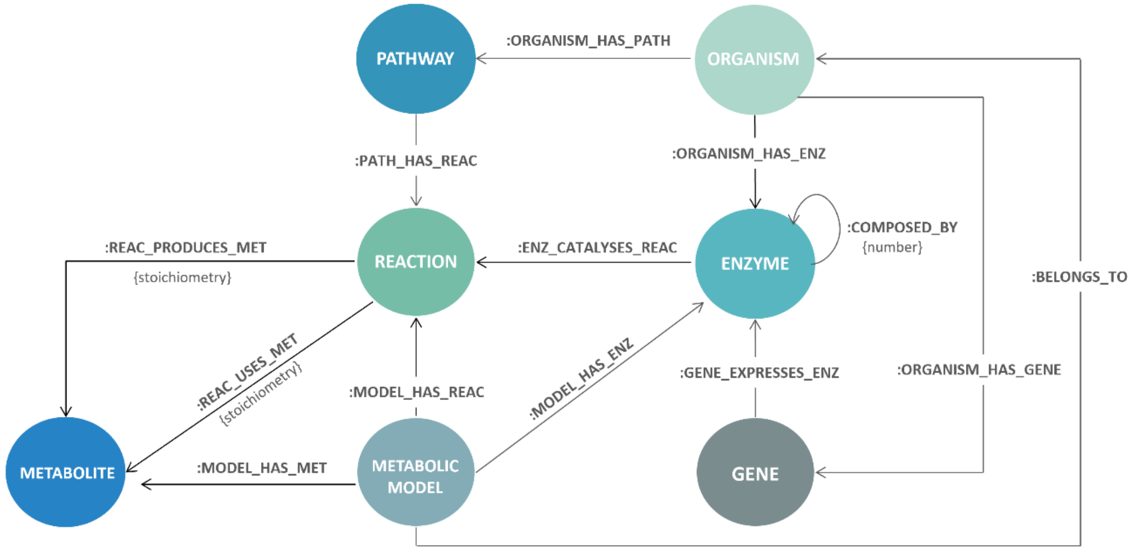

Fig. S1. Neo4j database schema representing the relationships between the data. It includes the nodes for metabolites, reactions, enzymes, pathways, genes, organisms, and metabolic models. The nodes are defined by four attributes: database identifier, name, timestamp, and database version, except for the metabolic model node, which includes a model identifier, organism, author, year, and timestamp. 13 relationship types are represented in the database: reactions are linked to metabolites by two different types of relationships, defining their reactants and products, to enzymes, and pathways. These two nodes and the genes are also linked to the organism node. Enzymes are also linked to genes and can be linked to other enzymes, in the case of protein complexes that are composed of protein monomers. Finally, the metabolic model node is linked to an organism and to metabolites, reactions, and enzymes, which define its content.

2. Model reconstruction

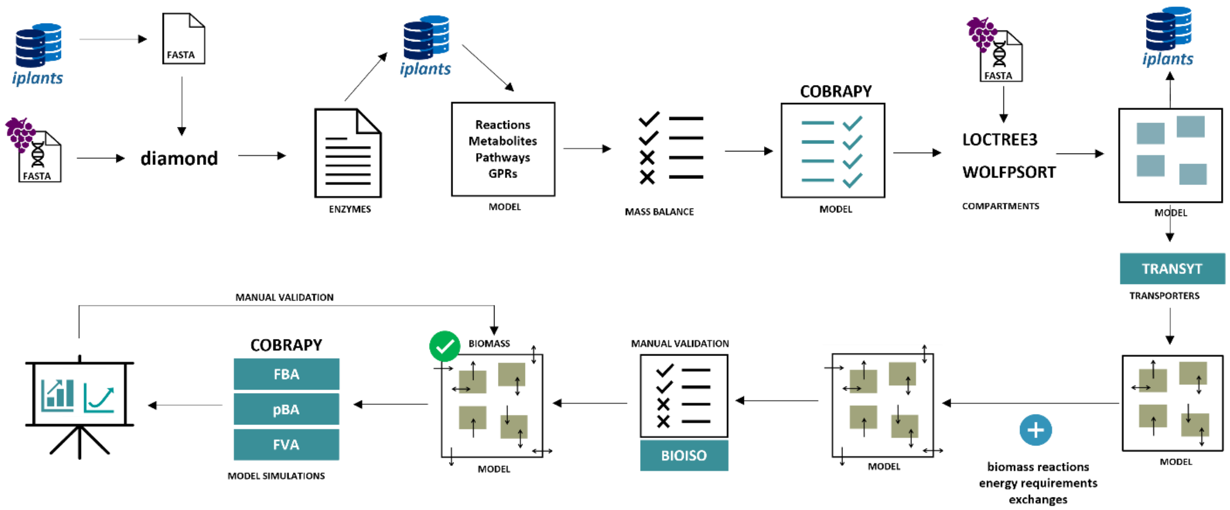

Fig. S2. The main steps of the *V. vinifera* GSMM reconstruction. Genome annotation is performed using DIAMOND with the protein sequences from the database and *V. vinifera* proteins from the genome. The identified enzymes are used to get all metabolic information from the database as well as to define the GPR rules in the model. Next, the mass balance of reactions is checked and fixed whenever possible. Unbalanced non-essential reactions were removed from the model. Then, subcellular compartments were predicted using LocTree3 and Wolfpsort tools, and transporters between these compartments were predicted using TranSyt. Finally, biomass, exchange, and energy requirement reactions were defined, and the model was manually validated, using BioISO to verify biomass production. The final model was simulated using different methods, such as FBA and FVA, and the results were analyzed to validate the model.

3. Model properties

3.1. Unique reactions

In total, *V. vinifera* has 785 reactions that are not present in any other model. These reactions were analyzed to identify the associated pathways and gene annotation. The pathways with more unique reactions are presented in Fig. S3. Reactions without pathway associations were not considered.

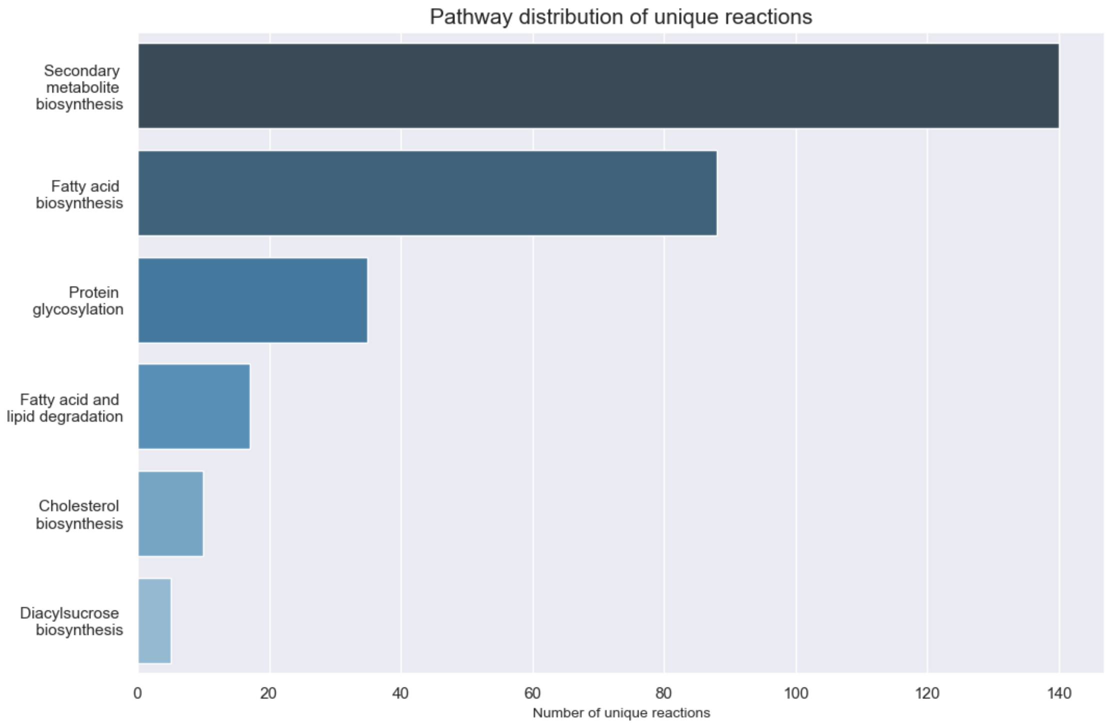

Fig. S3. Pathway distribution of reactions included in the *V. vinifera* model and not in the other plant models analyzed.

3.2. Specialized metabolic pathways

Secondary metabolites are economically very important as they have many relevant applications. However, the pathways that produce them are very complex and diverse, and the knowledge in this subject is still limited [1]. The production of the main secondary metabolites in the model, phenylpropanoids and terpenoids, are schematized in Fig. S4 and S5.

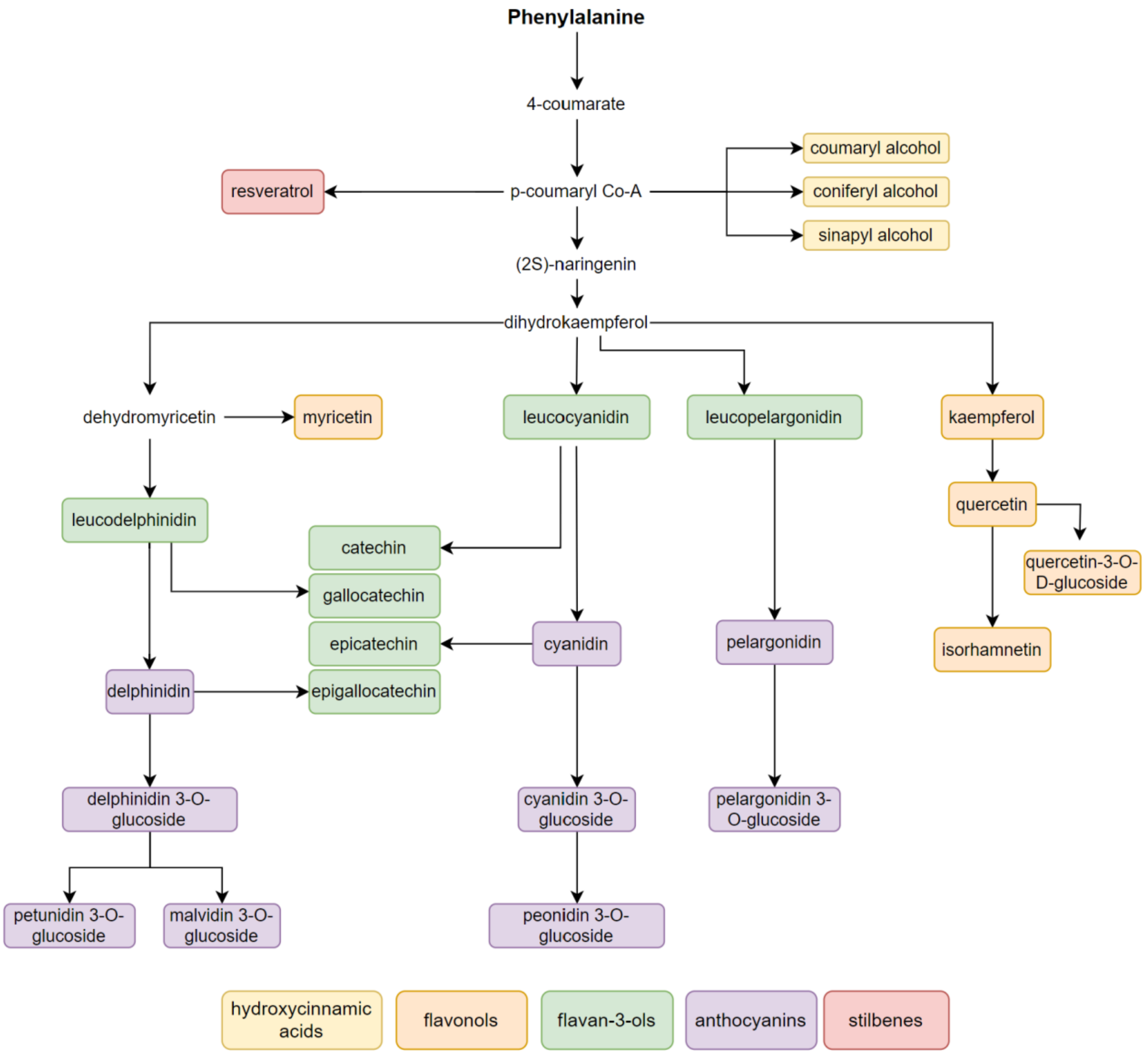

Fig. S4. Simplified schema of the phenylpropanoid pathway in the V. vinifera model. This pathway starts with the amino acid phenylalanine that is converted to p-Coumaryl Coenzyme A. This metabolite can be used to produce hydroxycinnamic acids, and stilbenes, such as resveratrol, or to start the flavonoid biosynthesis pathway to produce different types of flavonoids, such as flavonols, flavan-3-ols, and anthocyanins. Compounds are colored based on the compound class they belong to.

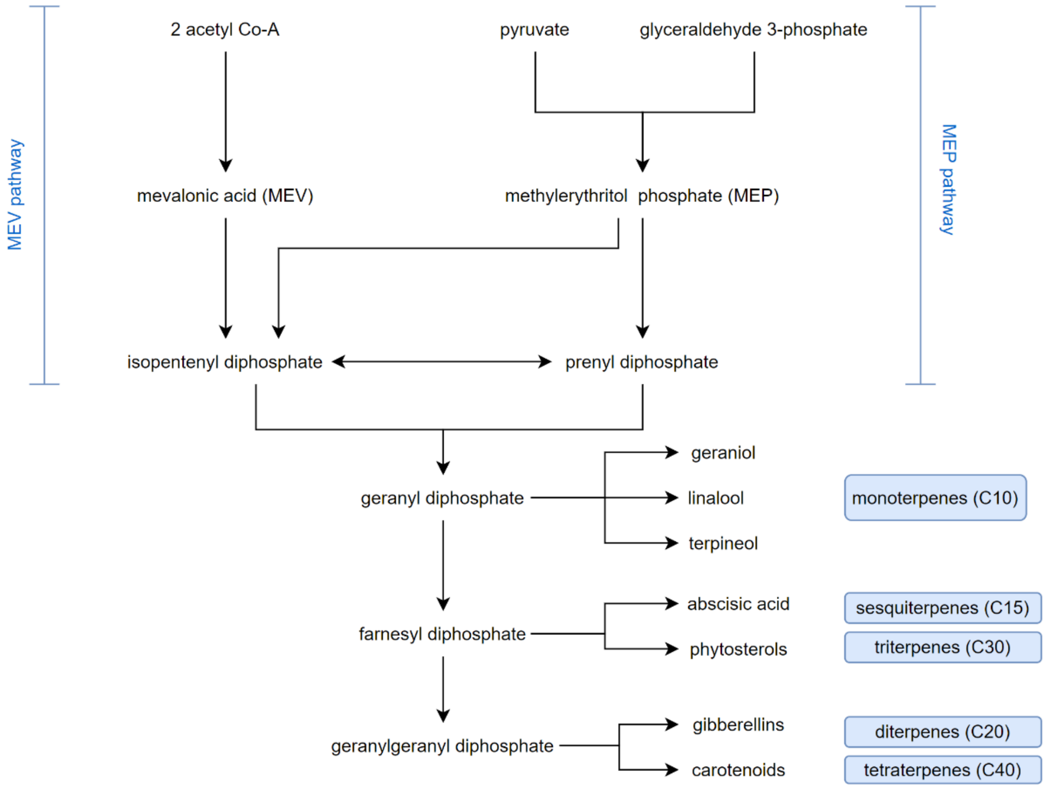

Fig. S5. Simplified schema of the terpenoid biosynthesis pathway in the V. vinifera model. Isopentenyl diphosphate (IPP) and prenyl diphosphate are the precursors for terpenoids and can be produced from the mevalonic acid (MEV) or methylerythritol phosphate (MEP) pathways. These originate all terpenoids including monoterpenes, sesquiterpenes, triterpenes, diterpenes, and tetraterpenes.

Grapes are known to have a high content of phenolic compounds and different grape varieties usually have different phenolic compositions, which leads to different wine flavours and aromas [2]. The reconstructed *V. vinifera* model contains complete pathways for the biosynthesis of several terpenoids and phenylpropanoids, which include flavonoids, such as quercetin, myricetin, kaempferol (and derivatives), and anthocyanins, like malvidin and peonidin. Anthocyanins usually accumulate during grape maturation and are responsible for the grape colour in red grapevine varieties, being absent in white varieties [3].

Another important group of phenylpropanoids in grapes are stilbenes, such as resveratrol, which protects grapes from intense UV light. As resveratrol is an antioxidant agent, it has high economic importance, being used in the pharmaceutical and cosmetic industry [4].

The complete pathway of resveratrol biosynthesis is described and included in the model, but there are many gaps in the biosynthetic pathways of resveratrol derivatives, such as viniferins, which are not included in the model but are important for wine flavour and aroma. However, this model also contains complete pathways for the biosynthesis of other aroma compounds, such as linalool, 1,3,5-trimethoxybenzene (TMB), and 3,5-dimethoxytoluene, the latter two being only described for *Rosa chinensis*.

In addition, complete secondary pathways for the biosynthesis of plant hormones, such as jasmonates, cytokinins, gibberellins, ethylene, and auxins, are available in the model. For instance, jasmonates are known to regulate seed germination and flower and fruit development, as well as to defend plants against some pathogens [5]. Cytokinins usually control cell growth and differentiation [6]. Although the reconstructed GSMM does not represent the action of these hormones, it can show the metabolic potential of the network to produce them.

Thus, the reconstructed model of *V. vinifera* represents an important source of secondary metabolic data. Further curation is still necessary to fill the existing gaps and increase the number of secondary metabolites in the model, as new knowledge on these pathways becomes available.

4. Tissue-specific models

4.1. Differential flux analysis

The ACHR sampler was used to generate 10000 sample fluxes for all reactions from the different tissue-specific models. Then, these data were used to identify the reactions with differential fluxes between models. In total, 764 reactions were found to have altered fluxes between at least two models. The complete flux dataset was then filtered to include only these reactions, scaled, and visualized with t-SNE. Hypergeometric enrichment tests were used to identify the pathways that presented significantly differential flux between each pair of models. These results are available in the Supplementary File 7.

Analyzing the results, it was clear that smaller pathways were not selected even when only one reaction was not identified as having differential flux. Therefore, this method seems to be more suitable for analyzing pathways with a large number of reactions. For this reason, the complete list of reactions with differential flux between the models was also analyzed.

Comparing the green and mature berry models, reactions from glycolysis, TCA cycle, and related to nucleotide biosynthesis were identified as having differential flux. In addition, anthocyanin biosynthesis exhibited more flux in the mature berry, as well as some reactions involved in the biosynthesis of quercetin and derivatives. This was expected as the mature berry has anthocyanins and a higher content of sugars in its biomass composition while having a lower content of nucleotides.

Nucleotide and anthocyanin biosynthesis pathways were also identified as having differential flux between the mature berry and the remaining tissues (leaf and stem). Other pathways included glycolysis and gluconeogenesis, photosynthesis, in the case of the leaf, and the pentose phosphate and gibberellin inactivation pathways in the case of the stem.

Comparing leaves with stem and green berry, the cellular respiration pathway was identified as having differential flux, as expected, mainly photosynthesis light reactions, Calvin cycle, glycolysis, gluconeogenesis, and pentose phosphate pathway. In addition,

the fluxes for the reactions of 4-aminobenzoate biosynthesis were also altered between these tissues.

In the case of stem and berry green models, besides glycolysis and pentose phosphate pathways, the reactions involved in folate metabolism, gibberellin inactivation, glutathione biosynthesis, and amino acid metabolism were also identified as having differential flux.

In summary, it was expected that the primary metabolic pathways would be identified as having differential flux between tissues, as tissue models have different demands for biomass precursors, and produce energy by different processes: the leaf performs photosynthesis, while the others perform aerobic respiration. Besides these, no relevant pathways were found to characterize the specific metabolism of each tissue. As mentioned before, most pathways in the model have few reactions, which makes it difficult to identify them using this method.

5. Diel multi-tissues:

Table S1. Fluxes for the metabolites stored between light and dark phases in the diel multi-tissue model with mature berry. Positive fluxes indicate that the metabolites are stored in the light phase to be used in the dark while the metabolites with negative fluxes are stored in the dark to be used during the day. The fluxes are in mmmol.gDW^{-1}.h^{-1}.

	reaction	flux
leaf	**CIT__vacu_leaf_light_dark_storage**	**-1.834**
	CYS__cyto_leaf_light_dark_storage	0.009
	ILE__cyto_leaf_light_dark_storage	0.024
	MAL__vacu_leaf_light_dark_storage	1.272
	MET__cyto_leaf_light_dark_storage	0.011
	**NITRATE__vacu_leaf_light_dark_storage**	**-1.059**
	PRO__cyto_leaf_light_dark_storage	1.143
	Starch__chlo_leaf_light_dark_storage	0.365
	SUCROSE__vacu_leaf_light_dark_storage	0.000
	THR__cyto_leaf_light_dark_storage	0.028
stem	**CIT__vacu_stem_light_dark_storage**	**-0.160**
	CYS__cyto_stem_light_dark_storage	0.003
	ILE__cyto_stem_light_dark_storage	0.008
	MAL__vacu_stem_light_dark_storage	0.044
	MET__cyto_stem_light_dark_storage	0.004
	SUCROSE__vacu_stem_light_dark_storage	0.027
berry	**CIT__vacu_berry_light_dark_storage**	**-0.021**
	CYS__cyto_berry_light_dark_storage	0.013
	ILE__cyto_berry_light_dark_storage	0.035
	MAL__vacu_berry_light_dark_storage	0.021
	MET__cyto_berry_light_dark_storage	0.016
	Starch__chlo_berry_light_dark_storage	0.011
	THR__cyto_berry_light_dark_storage	0.031

5.1. Sulfate assimilation

The *V. vinifera* green diel multi-tissue model was simulated to assess the effect of different sulfate concentrations on its metabolism. Flux Variability Analysis (FVA) was used to get the possible range of reaction fluxes while keeping at least 80% of the maximum total biomass value and fixing a photon uptake of 300 mmmol.gDW^{-1}.h^{-1}. Two different flux values for sulfate uptake were tested, 0.01 and 10 mmmol.gDW^{-1}.h^{-1}. The choice of these

values was arbitrary, but the goal was to have one value above and one below the unrestricted sulfate uptake flux.

Similar results were obtained for the multi-tissue with green and mature berries. Thus, only the results for the green multi-tissue are described. The full results are available in Supplementary File 9.

With high sulfate (10 $mmmol.gDW^{-1}.h^{-1}$), the maximum flux for biomass production decreased from 0.149 to 0.138 h^{-1}. Similarly, the production of all biomass components also decreased. As expected, the maximum fluxes of the reactions involved in sulfate assimilation and oxidation, and glutathione biosynthesis have increased. Surprisingly, in the model, the biosynthesis of cysteine and methionine decreased with high sulfate levels. During sulfate reduction, the reaction that produces H_2S has a higher maximum flux but the reaction that uses it to produce cysteine has a lower flux, which leads to a big increase in the flux of the H_2S exchange reaction (Fig. 4). This could mean that when plants are exposed to high sulfur levels, they try to adapt to these conditions by adjusting their metabolism, leading to the accumulation of H_2S or other sulfur compounds that can alter the flavor and aroma of the grapes.

In addition, the primary pathways carried less flux in high sulfur conditions, such as the TCA cycle, glycolysis, pentose phosphate pathway, and sucrose metabolism. The fatty acid metabolism has also decreased, as well as the metabolism of nucleotides, amino acids, and chlorophylls. Some photosynthesis light reactions presented a higher minimum flux. However, the downstream reactions from the Calvin cycle had lower maximum flux values as well as the aerobic respiration pathway (Fig. 4). The biosynthesis of secondary metabolites was also limited by higher levels of sulfur, which will also affect the flavor and aroma of grapes. As the biosynthesis of amino acids decreased, the maximum flux for amino acid storage also decreased with high sulfate levels.

When plants are under a sulfate deficiency, the production of biomass and all its components also decreases. For a sulfate uptake of 0.01 $mmmol.gDW^{-1}.h^{-1}$, the maximum production of biomass decreased from 0.149 to 0.018 h^{-1}. In addition, the biosynthesis of phosphopantothenate, cysteine, methionine, and coenzyme A, also decreased.

Hence, the plant has an excess of carbon and nitrogen skeletons, which are not being used for protein biosynthesis and are available for the synthesis of secondary metabolites, increasing the available flux for these pathways.

Therefore, there was an increase in the flux of primary pathways, such as sucrose and starch biosynthesis and degradation, gluconeogenesis, and glycolysis, as well as in the pathways responsible for producing secondary metabolites, plant hormones, and amino acids that are precursors of secondary compounds, like phenylalanine (Fig. 4). For instance, the maximum flux for resveratrol synthase reaction during the day increased from 0.41 to 1.75 mmmol.gDW^{-1}.h^{-1}. In addition, there was a great increase in the maximum flux for the storage of all amino acids, except for cysteine and methionine. Hence, sulfur levels in the soil can greatly influence grapevine metabolism and affect the flavor and aroma of grapes by sulfide or sulfur-compounds accumulation or changes in the phenolic content in grapes.

5.2. Nitrate assimilation

Nitrogen is one of the most important nutrients that plants need to capture from the soil, as it is a constituent element of several essential compounds, such as nucleic acids and proteins, thus being a limiting factor for plant growth, development, and survival. Previous studies have described that plants adapt to low availability of nitrogen by reducing photosynthesis, retarding growth, and accumulating anthocyanins, which are important compounds for grape and wine flavor and aroma. Hence, low nitrogen content is expected to increase anthocyanin production while decreasing plant growth [7,8]. A very high content of nitrogen is also expected to decrease growth and anthocyanin production, as the energy is redistributed to nitrogen assimilation.

The same approach was used to assess the effect of different nitrate concentrations in the *V. vinifera* model and similar results were obtained. Four different fluxes for nitrate uptake were tested: 0.1, 0.5, 5, and 10 mmmol.gDW^{-1}.h^{-1}. Although plants can also uptake nitrogen from ammonium, ammonium uptake was restricted to zero for simplicity.

For nitrate values of 0.1 and 0.5 mmmol.gDW^{-1}.h^{-1}, the maximum flux for total biomass, and to produce all its components decreased. For instance, the maximum flux value of

biomass decreased from 0.149 h^{-1} to 0.08 and 0.02 h^{-1} with a nitrate uptake of 0.5 and 0.1 $mmmol.gDW^{-1}.h^{-1}$, respectively. Conversely, the fluxes to produce fatty acids have increased. The maximum flux to produce fatty acids in the leaf during the day increased from 0.41 to 0.97 and 1.46 $mmmol.gDW^{-1}.h^{-1}$, with a nitrate uptake of 0.5 and 0.1 $mmmol.gDW^{-1}.h^{-1}$, respectively. Hence, as expected, the metabolism was redirected to the production of compounds that do not contain nitrogen (Fig. S6).

Other pathways with decreased flux include the biosynthesis of phosphopantothenate, 4-aminobenzoate, tetrahydrofolates, nucleotides, and some amino acids, like L-histidine, L-leucine, tryptophan, and L-ornithine. The maximum possible flux for storage between light and dark phases also decreased under lower values of nitrate uptake.

On the other hand, there was an increase in the flux of primary pathways, comprising sucrose and starch biosynthesis and degradation, gluconeogenesis, and glycolysis (Fig. S6). In addition, the pathways for DNA, RNA, uracil, glutathione, and amino acid degradation were increased to raise nitrogen availability. However, the biosynthesis of some amino acids was also increased, such as the biosynthesis of phenylalanine, which is the precursor for the phenylpropanoid pathway.

As described in the literature, most secondary metabolic pathways had an increase in the maximum flux, including the biosynthesis of phenylpropanoids (flavonoids, anthocyanins, stilbenes), isoprenoids, and gibberellins. The maximum flux of secondary reactions increased as nitrate uptake decreased. For instance, the flux for resveratrol synthase reaction during the day was 0.41 $mmmol.gDW^{-1}.h^{-1}$ in the unrestricted phenotype prediction and increased to 1.14 $mmmol.gDW^{-1}.h^{-1}$ when restricting the uptake of nitrate to 0.5 $mmmol.gDW^{-1}.h^{-1}$ and to 1.78 $mmmol.gDW^{-1}.h^{-1}$ with a nitrate uptake of 0.1 $mmmol.gDW^{-1}.h^{-1}$.

For high nitrogen uptake values (5 and 10 $mmmol.gDW^{-1}.h^{-1}$) (Fig. S6), most pathways presented lower maximum fluxes than in the unrestricted phenotype prediction, except for some reactions of photosynthesis, chlorophyll, tetrahydrofolate, and ATP biosynthesis, as well as the biosynthesis of some amino acids, such as histidine.

The maximum flux for the total biomass reaction decreased from 0.149 h^{-1} to 0.142 and 0.134 h^{-1} with a nitrate uptake of 5 and 10 $mmmol.gDW^{-1}.h^{-1}$, respectively. A decrease in the flux was observed in the producing reactions of all biomass components as well as most secondary pathways. The flux of the resveratrol synthase during the day slightly decreased from 0.41 to 0.39 and 0.36 $mmmol.gDW^{-1}.h^{-1}$ with a nitrate uptake of 5 and 10 $mmmol.gDW^{-1}.h^{-1}$, respectively. The maximum flux for the storage of some amino acids, such as serine and glutamate, decreased while it increased for others, such as valine and leucine. Although most pathways presented lower maximum fluxes with more nitrate uptake, the effects of low nitrate values appear to be more significant, as observed for sulfate.

No significant differences were found between the phenotype predictions of the green and mature diel multi-tissue models under low and high nitrate. The decrease in total biomass was slightly higher in the green berry model for both low and high levels of nitrate. Conversely, with low nitrate values, the increase in the maximum flux was slightly higher in the mature berry for most secondary reactions.

In summary, nitrate availability greatly affects plant metabolism, including secondary pathways. As for sulfate, controlling the levels of nitrate at certain stages of grape development is important to help control grape phenolic and sugar content, which will influence the flavor and quality of grapes.

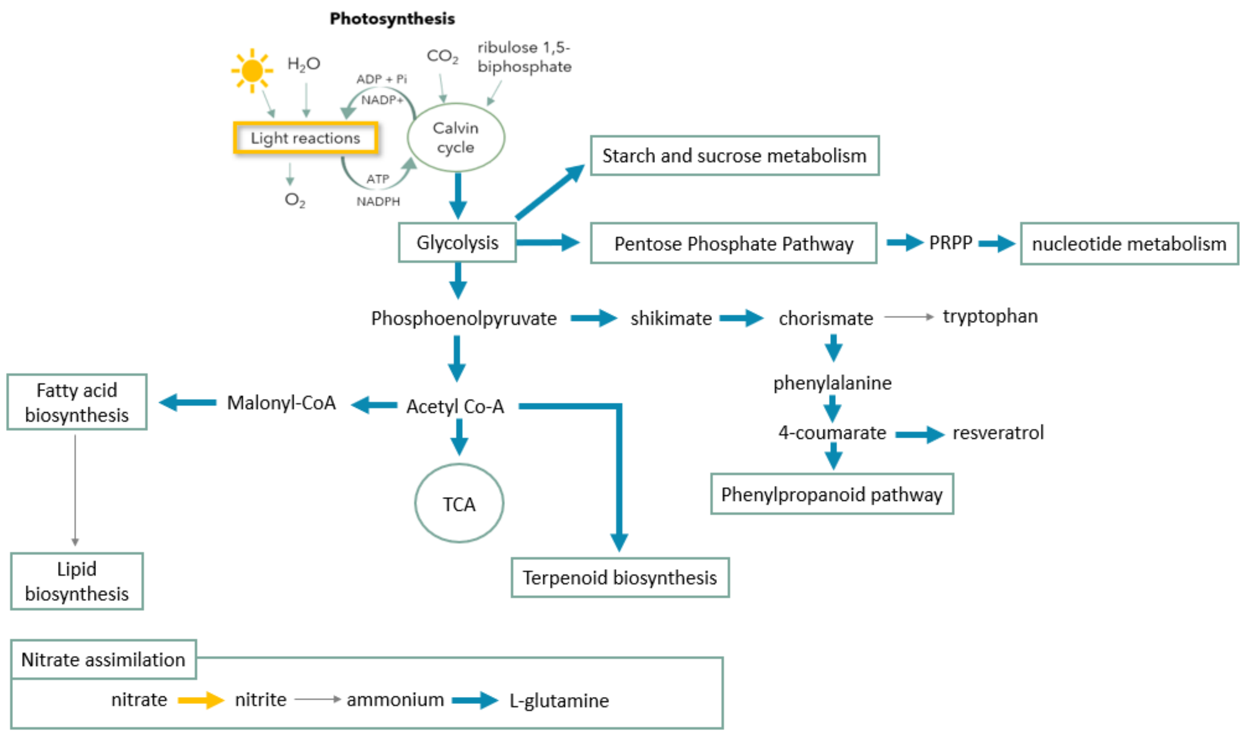

Fig. S6. Simplified schema of the main metabolic pathways in the model affected by varying nitrate levels. Pathways with increased maximum flux under low nitrate conditions are highlighted with a thick blue arrow while pathways with increased flux under high nitrate conditions are highlighted with a thick yellow arrow. Pathways with decreased flux in both cases are represented by a thin gray arrow.

6. Machine Learning and Fluxomics

6.1. Unsupervised analysis

First, data was preprocessed and explored using unsupervised methods, starting with t-SNE for data visualization. Before applying the t-SNE, the reactions with the same value in all samples were removed, which greatly reduced the dataset from 8632 to 2322 features. The results of the t-SNE are plotted in Fig. S7. A clear separation between the two states is shown as all green samples are located at the top of the plot while most mature samples are at the bottom. However, six mature samples were grouped with the green ones. These comprise Cabernet Sauvignon samples from time points 5 and 6 and Pinot Noir samples from time point 5. As veraison is expected to occur between time points 3 and 4, these results indicate that not many differences exist at the fluxomics level between green and early mature samples. Even so, fluxomics seems to distinguish well the remaining green and mature samples.

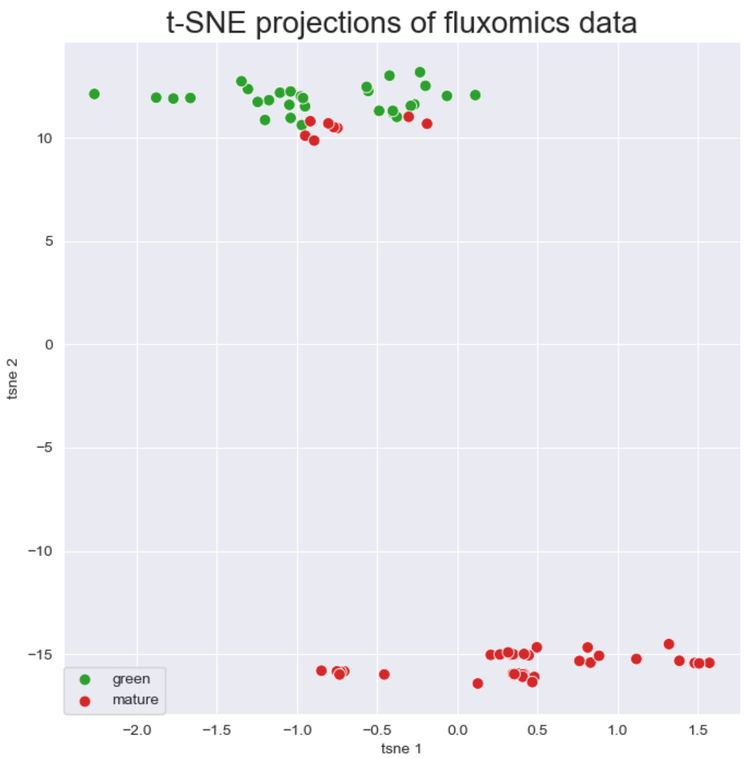

Fig. S7. t-SNE visualization of the fluxomics data obtained from the 73 context-specific GSMMs. Samples are coloured by the grape developmental stage.

6.2. Supervised analysis

SHAP values were calculated for the two best models, RF and KNN, trained with fluxomics data generated from *V. vinifera* models.

The most contributing reactions are shown in Fig. S8 for KNN and Fig. 5 for RF.

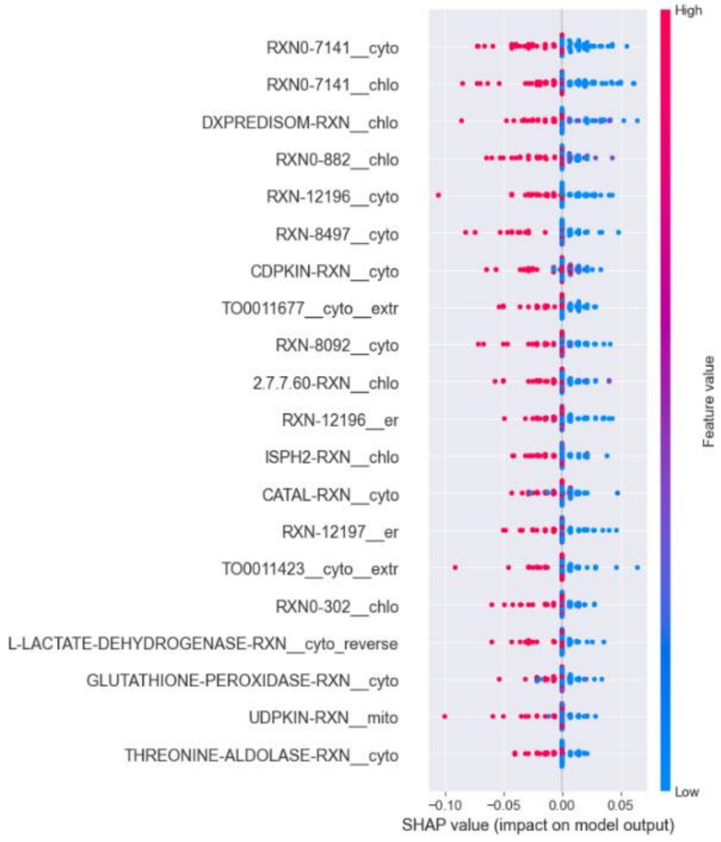

Fig. S8. Beeswarm plot of SHAP values for the reactions that contribute most to KNN's predictions. Features are ordered from higher to lower effects on the predictions. The dots represent a single observation, and the color indicates if the observation has a higher (pink) or a lower (blue) feature value compared to the other observations.

Overall, when the reactions presented high flux capacity (FCa) values they had negative SHAP values, meaning that they had a negative contribution to the model's predictions, while with lower FCa they exhibited higher positive SHAP values. This means that high fluxes of these reactions lead the model to predict the green state, while lower fluxes lead the model to predict mature states. Hence, all the reactions identified here presented an average FCa value higher in the green samples than in the mature grapes.

For the KNN model, the RXN0-7141 in the cytosol has the highest contribution to the predictions. High fluxes of this reaction have a negative contribution (SHAP value lower than -0.05), while lower fluxes have positive SHAP values (SHAP value higher than 0.05). Hence, high fluxes of this reaction are associated with the green state while low fluxes are linked to the mature state.

For the RF model, RXN0-882 in the chloroplast is the reaction that most contributes to the predictions. Similarly, high fluxes of this reaction have a negative impact on the model (SHAP value around -0.15), classifying the samples as green, while lower fluxes have a positive impact (SHAP value close to 0.10), classifying the samples as mature. There are some exceptions to this trend, such as the chloroplastic THREONINE-ALDOLASE-RXN and THRESYN-RXN reactions that show positive SHAP values when presenting high flux for the RF model, indicating that samples classified as mature by the models can also have high fluxes for these reactions.

Of the 20 reactions identified for each model, 10 have a high impact on both models, indicating that the results are reliable and robust and that these features are important for predicting the output. Most of these reactions are involved in the methylerythritol phosphate (MEP) pathway, which is responsible for the biosynthesis of the terpenoid precursors. The other reactions in common are associated with threonine degradation into glycine (THREONINE-ALDOLASE-RXN), and the transport of glycerides (TO0011677, TO0011423).

The reactions identified only for the KNN model are related to several different pathways including fermentation (L-LACTATE-DEHYDROGENASE-RXN), the degradation of triphosphate nucleotides (RXN-12196 and RXN-12197), reactive oxygen species (CATAL-RXN and GLUTATHIONE-PEROXIDASE-RXN), and ethanol (RXN-8092), and the biosynthesis of fatty acids (RXN-8497) and nucleotides (CDPKIN-RXN and UDPKIN-RXN).

Analyzing the reactions identified only for the RF model, RXN66-3 is similar to the reaction RXN-8092 already identified for KNN. The remaining reactions are also involved in the MEP pathway (2.7.1.148-RXN and RXN0-884), the biosynthesis of jasmonate derivatives (RXN-10435), nucleotides (RXN-14325), 4-aminobenzoate (ADCLY-RXN and PABASYN-

RXN), and the homoserine and threonine amino acids (THRESYN-RXN, ASPARTATEKIN-RXN, HOMOSERDEHYDROG-RXN).

The accumulation of terpenoids in grapes typically starts before veraison, which can explain why the reactions associated with the biosynthesis of terpenoid precursors had higher FCa in the green state. However, terpenoid biosynthesis intensifies after veraison, which is not observed in the fluxes of these reactions. Fasoli et al. [9] has also identified terpene metabolism as a negative biomarker for the onset of ripening. In addition, the abscisic acid (ABA) signaling is increased at veraison, and ABA is derived from carotenoids, whose biosynthesis starts with the MEP pathway. Thus, there is strong evidence that genes or reactions from the MEP pathway could be used as biomarkers for the onset of ripening.

In the green phase, as grapes are rapidly growing, the metabolism of amino acids, nucleotides, lipids, and oxidative stress is expected to be more active than in the mature phase. 4-aminobenzoate is a precursor for the biosynthesis of various metabolites, such as tetrahydrofolates, which are involved in several processes like photorespiration, amino acid metabolism, and protein biosynthesis. These pathways are also expected to be more active in the green phase. This fact may explain why the reactions related to these pathways are important for the model's predictions. However, it is not clear why threonine metabolism is more important for the model than the metabolism of the other amino acids.

Although most of the reactions that have a higher contribution to the model's predictions are associated with pathways that appear to be more active in the green phase, these pathways were not the most expected as the major differences between green and mature grapes are their content in organic acids, sugars, and phenolic compounds. This could be due to the limitations in the metabolic models, mainly in the GPR rules or biomass definition, which forces the production of some biomass precursors to allow the simulations, reducing the difference between the fluxes of these reactions in the different phases.

Nevertheless, the models presented good predictions, associating high fluxes of these reactions to the green state and low fluxes to the mature state.

References

1. Collakova E, Yen JY, Senger RS. Are we ready for genome-scale modeling in plants? Plant Science. 2012;191–192: 53–70. doi:10.1016/j.plantsci.2012.04.010

2. Singh J, Kumar M, Sharma A, Pandey G, Chae K, Lee S. Phenolic Compounds of Grapes and Wines: Key Compounds and Implications in Sensory Perception. Intech. 2016.

3. Massonnet M, Fasoli M, Tornielli GB, Altieri M, Sandri M, Zuccolotto P, et al. Ripening transcriptomic program in red and white grapevine varieties correlates with berry skin anthocyanin accumulation. Plant Physiology. 2017;174: 2376–2396. doi:10.1104/pp.17.00311

4. Saad NM, Sekar M, Gan SH, Lum PT, Vaijanathappa J, Ravi S. Resveratrol: Latest scientific evidences of its chemical, biological activities and therapeutic potentials. Pharmacognosy Journal. 2020;12: 1779–1791. doi:10.5530/pj.2020.12.240

5. Wasternack C, Song S. Jasmonates: biosynthesis, metabolism, and signaling by proteins activating and repressing transcription. Journal of Experimental Botany. 2017;68: 1303–1321. doi:10.1093/JXB/ERW443

6. Kieber JJ, Schaller GE. Cytokinins. The Arabidopsis Book / American Society of Plant Biologists. 2014;12: e0168. doi:10.1199/TAB.0168

7. Yin H, Li B, Wang X, Xi Z. Effect of ammonium and nitrate supplies on nitrogen and sucrose metabolism of Cabernet Sauvignon (Vitis vinifera cv.). Journal of the Science of Food and Agriculture. 2020;100: 5239–5250. doi:10.1002/JSFA.10574

8. Liang J, He J. Protective role of anthocyanins in plants under low nitrogen stress. Biochemical and Biophysical Research Communications. 2018;498: 946–953. doi:10.1016/J.BBRC.2018.03.087

9. Fasoli M, Richter CL, Zenoni S, Bertini E, Vitulo N, Dal Santo S, et al. Timing and order of the molecular events marking the onset of berry ripening in grapevine. Plant Physiol. 2018;178: 1187–1206. doi:10.1104/pp.18.00559
