# Supplementary material for: A diel multi-tissue genome-scale metabolic model of Vitis vinifera
Source: PLoS Comput Biol. 2024 Oct 10;20(10):e1012506. doi: 10.1371/journal.pcbi.1012506 (PMC11495577; doi:10.1371/journal.pcbi.1012506)
Supplement: Attachment — Submitted filename: Reviewers_answers_final.pdf [file pcbi.1012506.s011.pdf]

Reviewer's Responses

- **Reviewer #1:**

General comment:

In this manuscript, Sampaio et al. present the reconstruction of the genome-scale metabolic model of the plant Vitis vinifera. Their aim is to reconstruct a diel multi-tissue model capable of reproducing the main metabolic landscape of this organism. I appreciate the effort that the authors made in reconstructing the model and in including many specific pathways that were not covered in previous reconstructions. Also, they made extensive use of manual curation, which is good. I loaded the model and ran a few basic simulations and everything worked correctly. Surely, the model will be a valuable contribution to those working in the field of plant systems biology and/or functional genomics. The authors show that their model is more comprehensive than other currently available plant models and, as such, it is a nice addition to the actual literature. Apart from this strong effort in the reconstruction phase, the manuscript lacks a bit in originality and does not provide many new and/or innovative aspects of plant metabolism.

Author response:

First, we thank the reviewer for the insightful comments on our manuscript. We appreciate the recognition of our extensive effort in reconstructing the genome-scale metabolic model of *Vitis vinifera*.

We acknowledge the constraints on the originality and innovative aspects of our study. Such limitation is largely due to the limited knowledge on *Vitis vinifera's* plant metabolism and the current scarcity of comprehensive public plant omics datasets that would allow more relevant applications of our model. Nevertheless, we believe our model provides a valuable framework that can be further refined and expanded for more innovative applications as more data becomes available.

Also, the reading of the manuscript is quite tough. The text is too long and quite hard to follow, especially in the first part. I would encourage the authors to present most of the text and figures of pages 10 to 14 (surely figures 3 and 4) as additional material. This would make the manuscript easier to read and allow the authors to focus most on the use of the model rather than its assembly. There might be other sections that could be presented as additional material in the manuscript so my suggestion is to go through the manuscript once more and try to reduce the text (and maybe figures) to improve the overall readability.

Author response:

As the model was the main output of our work and the manual curation of secondary pathways required the most effort, we included it in the paper. However, we agree that the paper is too lengthy, so we have followed the reviewer's suggestion and moved the section on *Specialized metabolic pathways* to the supplementary material.

I also think that the authors could use the model in a more "propositive" and innovative way to explore some key issues of plant metabolism. A few examples that the authors might want to take into consideration.

Could the grape models at different stages of maturations be used to infer which are the key pathways that affect (or maybe start) the process of grapes rotting?

Could the same models be used to identify targets that could be exploited to improve and/or modify the metabolic content of grapes and, as a consequence, the taste of wine?

Author response:

We appreciate the reviewer's interesting suggestions for possible innovative applications of the model.

However, regarding the first suggestion, without omics data from rotted grapes, it is impossible to identify the pathways involved in that process. We investigated the differences between grape models at different stages of maturation and have identified the main active pathways at different stages. Although the results did not yield new insights into grape metabolism, this was the most comprehensive analysis possible with the available omics dataset. We, too, wished for more data to expand our findings.

Regarding the second suggestion, it is known that the metabolic content of grapes is affected by its content of sugars, organic acids and phenolic compounds. Mainly, any step in the phenylpropanoid pathway could be a potential target to modify the metabolic content of grapes. In addition, as we described in the paper, the levels of sulfur and nitrogen in the soil also influence the metabolic content of grapes as they interfere with the central metabolism and, consequently, with the metabolic flux that go through the secondary pathways. Hence, for a more comprehensive analysis of the metabolic pathways impacting grape metabolic content, omics data from both high-quality and low-quality grapes should be integrated with the model to determine which pathways contribute to each phenotype. Another approach would be to define an ideal grape metabolic profile and use the model to achieve this profile, identifying the key pathways involved. However, defining "improved metabolic content of grapes" is challenging, as different wines require different flavor profiles. Although our model has a wide range of potential applications, it is currently limited by the lack of public plant metabolic data.

Specific points:

<u>Point 1:</u>

Line 150 (and elsewhere?): (Fig. 1Error! Reference source not found.). Please correct.

Author response:

We thank the reviewer for noticing this error in the figure references. We fixed that.

<u>Point 2:</u>

Lines 154 - 158: Do these differences in the number of reactions/genes/metabolites reflect the evolutionary differences between the species or simply reconstruction efforts?

Author response:

These differences reflect both the evolutionary differences between the species and the reconstruction efforts. Since the reconstruction is based on the genome of each organism, evolutionary variations are inherently captured. However, the results are also influenced by the amount of metabolic knowledge available at the time the models were reconstructed.

<u>Point 3:</u>

Line 299 (and other): "The leaf tissue was simulated for all processes". I think here the authors meant "The flux distribution in the leaf was simulated ...". I advise the use of this form throughout the text.

Author response:

We thank the reviewer for pointing this out, and we changed the text for clarification.

<u>Point 4:</u>

Line 301: "Photosynthesis can also occur in green berries, but not at significant levels". A reference is needed here.

Author response:

We thank the reviewer for noticing the missing reference and we added it to the text.

<u>**Point 5:** typo?</u>

Line 336: -> "Among models"

Author response:

We thank the reviewer for this suggestion, and we changed the text.

<u>Point 6:</u>

Line 506: "When plants are under a sulfate deficiency, the production of biomass and all its components also decreases." Sulfate likely participates in many reactions of the model and is probably included in the biomass reaction (coenzymes?), thus it is not surprising that reducing its uptake from the environment lower biomass is produced and fluxes are generally low.

Author response:

Yes, we agree with the reviewer that the results were expected. This statement was included to clarify why the flux increases in the secondary pathways under sulfate deficiency.

Point 7:

Line 524: Machine Learning and Fluxomics paragraph. In this section it is not clear that fluxomics data is coming from constraint-based simulations. Please make it clear early in the paragraph that this is the case.

Author response:

We accept the reviewer's suggestion and have revised the text to clarify that the fluxomics data are derived from constraint-based model simulations.

Reviewer #2:

General comment:

The manuscript "A diel multi-tissue genome-scale metabolic model of Vitis vinifera" by Sampaio et al. describes (i) the iPlants repository, a collection of relevant data for reconstruction of metabolic data and also nine published and publicly available plant metabolic models; (ii) the reconstructed GSMM of V. vinifera and compared the reaction contents of their model with other seven plant metabolic models; (iii) pathway distributions of the reactions present in their model but not in other models; (iv) integration of RNA-seq data with the GSMM to get tissue specific models; (v) diel-multi tissue (leaf, stem, berry) model; (vi) how the machine learning method is used to analyze flux data of different content-specific GSMMs. Further, some simulation results were shown to match with known biochemical active pathways in specific condition.

However, I cannot recommend to accept the manuscript in its present form due to following reasons:

1. *The authors claim in their abstract that "advances have been made, allowing the integration of omics datasets with GSMMs". It is not clear in the manuscript what is the specific advancement achievement in this paper. Integration of omics data as well as multi-tissue modeling was reported earlier. One such examples is "Reconstruction of Arabidopsis metabolic network models accounting for subcellular compartmentalization and tissue-specificity. https://doi.org/10.1073/pnas.1100358109"*
   Nadine Töpfer, Camila Caldana, Sergio Grimbs, Lothar Willmitzer, Alisdair R. Fernie, Zoran Nikoloski, Integration of Genome-Scale Modeling and Transcript Profiling Reveals Metabolic Pathways Underlying Light and Temperature Acclimation in Arabidopsis , The Plant Cell, Volume 25, Issue 4, April 2013, Pages 1197–1211, https://doi.org/10.1105/tpc.112.108852 . Interestingly, these types of breakthrough relevant works have not been cited/discussed in this current manuscript. Thus, it is incomplete.

Author response:

First, we want to thank the reviewer for the comments and suggestions. In the abstract, we mentioned that advances have been made in the last years regarding the genome scale metabolic modelling of plants and omics integration to justify the application of these approaches to study grapevine metabolism. The advances were achieved in prior works, including the manuscripts described by the reviewer. The main novelty of our study is the application of these methos to an important crop plant as *Vitis vinifera*. Given the inherent challenges of plant genome-scale metabolic model reconstruction, we focused on creating a comprehensive metabolic network. Although the available approaches for multi-tissue genome-scale metabolic models reconstruction and omics integration are not flawless, we believe that the main limitation is the lack of omics data and knowledge on plant metabolism, which can lead to incomplete metabolic models. We acknowledge the importance of the works mentioned by the reviewer and have cited them in the introduction, on line 66.

2. *The authors state "Furthermore, to capture the dynamic changes in grape metabolism, we created two separate models representing the grape in both its green and mature states." I have not found any section that clearly explains/describes the dynamic changes they have been able to capture.*

Author response:

We agree with the reviewer that this phrase could be misleading. We aimed to capture dynamic changes in grape metabolism by analysing the differences in the reaction fluxes of the grape in the green and the mature model, which are descried in the differential flux analysis. Unfortunately, the differences between the two states were not very pronounced, reflecting mainly the differences in the biomasses of the models representing each grape state. This suggests that the average gene expression values for green and mature grapes did not result in relevant differences in the fluxes of the reactions.

Therefore, we remove the word "dynamic" of the text.

3. *The manuscript is very badly represented. For example, the limitations like Line 273: "The biomass of leaf and green berry was considered to be the same." should be discussed separately in a subsection. Whether they have used any maintenance cost in the model is not clear. Many figures (Figures 2,3,4) may be removed to supplementary.*

Author response:

We agree with the reviewer that this phrase is unclear. We assume the same biomass coefficients for leaf and green berry due to the lack of specific biomass data for *V. vinifera*. We based these values on available plant models, but most available models only represent the leaf tissue, and some include the stem. Therefore, it was challenging to decide how to represent the biomass for the berry. For simplicity, we kept the biomass of the green berry equal to the leaf and defined the biomass of the mature berry based on the differences to the green berry described in the literature. This approach is not ideal, but these values can be easily updated when biomass data for *V. vinifera* becomes available.

We also appreciate the reviewer for noticing that information on maintenance cost was missing in the paper and we added the following paragraph to the biomass composition section in the materials and methods:

As no information regarding the energetic requirements for V. vinifera was found in the literature, it was retrieved from the model of S. lycopersicum [35]. The growth-associated maintenance energy requirement was included in the biomass equation and defined as 53.26 mmmol.gDW^{-1}. The nongrowth-associated maintenance energy requirement was defined in an ATP hydrolysis reaction with a mandatory flux of 2 mmmol.gDW^{-1}.h.

We also accept the reviewer's suggestion to move the figures 2, 3, and 4 to the supplementary material.

Reviewer #3:

General remarks and main concerns:

In this manuscript, the authors reconstructed a generic genome-scale metabolic model of grapevine metabolism. They explore the metabolism with a model combining tissue-specific models based on omics data of stem, leaf, and berry, and day-night modes. Fluxes were analysed by ML. The manuscript is written relatively clearly and the work carried out is substantial in terms of bioinformatics tools used for the reconstruction of the model, but the flux analyses and the ML methods used (t-SNE) are poorly justified and not always relevant.

1. *The biggest concern is that the reconstructed model is proven to account for physiology by taking very coarse and global data, such as for example the same biomass composition for the leaf and the young berry, a protein content of 60% in the mature berry (huge and higher than young berries) or the same growth rate for all tissues. This information cannot properly account for flux predictions in line with real phenotypes. Even if there are no data for grapevines, it would be preferable to take tissue-specific data from another plant. This crude parameterization can largely explain the lack of phenotype difference in the predicted fluxes.*

Author response:

We agree with the reviewer that the biomass definition is very important for obtaining more realistic flux predictions. The lack of experimental biomass data is a significant limitation of the study, as it is necessary to create accurate tissue-specific models. We did indeed define the biomass composition using tissue-specific data from other metabolic models of plants. However, most of the available models only consider the leaf tissue, with some including the stem. Therefore, the differences between the leaf and stem biomass compositions are the same observed in other plants models.

The main challenge was to define the berry compositions. For simplicity, we assumed that the the biomass of the green berry would be equal to the leaf and then defined the biomass composition of the mature berry based on the grape metabolic content measurements from the literature. Although this is not ideal, we believed that the omics dataset would restrict the model enough to reveal the main differences between the metabolism in green and mature berries metabolism. Unfortunately, our results did not confirm this.

Nevertheless, the model can be easily updated with real biomass composition values when data of *V. vinifera* become available, making it a valuable tool for analysing *V. vinifera* metabolism. We used the same growth rate to focus our analysis on the main metabolic differences between tissues.

We acknowledge that we did not analyse the metabolic changes in grape during development, but rather compared the metabolic content of green and mature grapes. Again, the differences between the two states were not very evident, reflecting mainly the differences in the biomasses of the models representing each grape state. This suggests that the average gene expression values for green and mature grapes did not result in relevant differences in the fluxes of the reactions. We described only the reactions with

differential flux to highlight the main differences in the simulations of the different models, although we analysed all reactions, and found the fluxes to be very similar.

2. *Also, the multi-tissue model is reduced to 2 stages of berry development, so it is inappropriate to talk about metabolic changes during development. And beyond the separation of tissues and the 2 stages, on the basis of differential flows, the model does not provide relevant physiological conclusions. Why are only differential flows looked at? Also, the ML method used, t-SNE, is not justified, what does it offer compared to a more traditional PCA? why not look for the variables explaining the separations?*

Author response:

We acknowledge that our multi-tissue model comprises only two stages of berry development, this is because we focused on comparing the metabolic content of green and mature berries as the available data only allowed to do so. We chose to analyse differential flows to highlight the main metabolic differences between the tissues and stages, which could potentially suggest for instance, (de)activation of specific pathways.

Regarding the ML method, we selected the t-SNE instead of PCA because t-SNE can establish non-linear relationships between features and highlight the clusters that are close together. This is particularly advantageous for visualizing complex high-dimensional data like omics and flux distributions.

3. *As for the day-night model, whether for young or ripe berries, the phenotype predictions (table 4) are very similar; It's surprising and disappointing. Also, an equivalent flux of biomass is impossible because the mature berry is no longer growing, unlike the young berry.*

Author response:

We agree with the reviewer that the results of the diel simulations did not show relevant differences between green and mature berries, which was expected given the lack of evident differences in the active pathways of each grape model. The constraints defined from the omics data of green and mature grapes were not sufficient to create relevant differences in the flux simulations of both states. Additionally, the diel models included all grape tissues, not only the berry, which also increased the complexity of the network.

We assume the same light uptake value to qualitatively compare the active pathways of the two models. For obtaining more realist results, the light uptake or the biomass production can and should be constrained to more realistic values.

Finally, we acknowledge that an equivalent flux of biomass is not feasible because the mature berry is no longer growing, unlike the young berry. This is a limitation of our current model setup, which can be addressed in future studies with more detailed and accurate data.

4. *Finally, the last part using ML on fluxomics data is complex and does not clearly highlight the underlying results obtained whether with t-SNE or with the predictions. For the interpretation part of the variables underlying the predictions, why is it mentioned that 10 of the 20 reactions have a strong impact? Should the same reactions have a strong impact for all 4 models?*

Author response:

As no significant differences were found between the flux distributions of the green and mature grapes, we applied a different approach to compare both states. In this case, a model was created for each sample of the omics dataset. Therefore, these results are not comparable to the flux simulations obtained using only one model to represent each grape state.

All machine learning models obtain good metrics when comparing both states. The fact that certain reactions had a high impact on classification across different machine learning models, suggests that the results are robust and independent of the specific model used. This indicates with higher confidence that these 10 reactions are important for classifying a grape as green or mature.

5. *Why did the authors have to calculate flow capacity, in addition to flux estimates?*

Author response:

We decided to compare the simulations using the reactions flux capacity because a flux estimate represents only one possible flux value within a solution space. Different models can originate similar flux simulations but have different flux capacities defined by the constraints. For instance, a reaction producing a secondary metabolite might be blocked in the green berry due to a constraint defined by the omics dataset, resulting in a flux estimate of zero. In the mature berry, the same reaction might have a flux estimate of zero not because it is blocked but because the metabolite is not needed for biomass production. In this case, the flux capacity of that reaction will be different than zero in the mature state and different from the green state. Comparing flux capacities helps us understand these underlying differences more clearly.

6. *Finally, there are many figures (12) and some are not very relevant for this work (figs 3 and 4) or not very useful, like fig 5. There are also many tables (actually 6). Some of them are difficult to understand such as Table 5 with the names of the reactions are not explicit.*

Author response:

We agree with the reviewer that the paper is quite lengthy. We included several figures and tables to facilitate following all the different analyses performed in the work. However, the large number of images and tables may indeed make the paper difficult to read. Therefore,

we have decided to remove the figures 3,4 while keeping Figure 5, as it compares the t-SNE of the RNA-Seq data with the t-SNE of the fluxes, which we believe is important to illustrate our analysis.

Regarding Table 5, the names of the reactions are the identifiers used in the model. To improve clarity, we have simplified these names.

Minor concern:

Point 1:

L480: why is the FVA done here and not before?

Author response:

In the differential flux analysis, we decided to use flux sampling instead of FVA, as it was previously described as a good approach to compare flux distributions from different tissue-specific models (Nanda P, 2021). Here, we use FVA to better analyse the effects of the different sulfate concentrations on the flux distributions.

Point 2:

L 788: the nitrate uptake was constrained to a ratio of 3:2, in terms of what?

Author Answer:

Nitrate uptake in the light was one third higher than in the dark. This ratio was based on available diel plant models.

Point 3:

l 271, l 398 and later: typography problem to cite tables

Author Answer:

We thank the reviewer for noticing this problem and we fixed it.

Point 4:

Legend fig 5 is not "RNA-Seq data for all tissues" but the percentage of RNA-Seq analyzes for all tissues.

Author Answer:

We accept the suggestion, and we changed in the text.

Point 5:

Legend fig 6 is not “Biomass composition” but percentage (?) of the main biomass compound in the biomass.

Author Answer:

We accept the suggestion, and we changed in the text.
