# Supplementary material for: A diel multi-tissue genome-scale metabolic model of Vitis vinifera
Source: PLoS Comput Biol. 2024 Oct 10;20(10):e1012506. doi: 10.1371/journal.pcbi.1012506 (PMC11495577; doi:10.1371/journal.pcbi.1012506)
Supplement: Attachment — Submitted filename: Reviewers_answers_pt2.pdf [file pcbi.1012506.s012.pdf]

Reviewer's Responses

- **Reviewer #1:**

I acknowledge the effort that the authors made in reconstructing such a large and complex model. I think it will be a valuable resource in the next years.

Author response:

First, we thank the reviewer for the insightful comments on our manuscript. We appreciate the recognition of our extensive effort in reconstructing the genome-scale metabolic model of *Vitis vinifera*.

At the same time, I feel like the authors could/should have put more effort in simplifying and shortening the text. The revised version has been reduced by approx. 1 and a half page.

Author response:

We agree that our manuscript is still too long and decided to simplify the document. For such, we removed the t-SNE analyses of the RNA-Seq data and the tissue-specific sampled fluxes of the differential flux analysis section. The t-SNE analysis of the fluxomics data from the Machine Learning and Fluxomics section was moved to Supplementary File 1.

Also, I am not entirely convinced by the fact that there are no sufficient -omics data to interrogate the model further and provide more insights into the metabolism of this plant in disparate conditions. V. vinifera is one of the best studied plants and there are many interesting RNAseq datasets in the databases. I am not saying that the authors should add some additional analyses here but it would be nice to have a sentence or two on the possible use of the reconstruction to investigate some specific metabolic phenotypes of V. vinifera in the future.

Author response:

Regarding omics datasets, in Gene Expression Omnibus (GEO) database there are around 30 datasets with *V. vinifera* gene expression profiles obtained from high-throughput technologies. Most of these datasets are from grape samples and not many studies were available to define the leaf and stem tissues. Also, the main limitations of these datasets are the low number of samples that prevent the use of machine learning approaches to classify the samples and identify new biomarkers. In addition, most omics studies comparing the gene expression in *V. vinifera* under

different environmental conditions or diseases were performed using microarrays. Given the limitations of this technology, we decided to use only RNA-Seq data.

Following the reviewers' suggestions, we decided to include a conclusion section with the limitations of the current work and the possible applications of the GSMMs of *V. vinifera* when more data becomes available. Other applications of these models include the study of grapevine metabolic responses to environmental stresses, such as drought, or to understand disease mechanisms. Omics data of resistant and susceptible grapevines under an environmental stress, or healthy and infected grapevines in the same environment are necessary to perform these analyses.

The following text was added to the conclusion section:

"For instance, in future work, the model can be integrated with other datasets to investigate condition-specific metabolic phenotypes, such as responses to environmental stresses, like drought, or to understand disease mechanisms and developmentally regulated pathways, thereby offering a more comprehensive understanding of the plant's metabolism."

- **<u>Reviewer #2:</u>**

The authors have tried to address the queries and suggestions of myself and other reviewers. However, I think that a different subsection describing the limitations of the present study and how these limitations do not affect the conclusion of the present work is very much needed. It is even clear from the author's response to reviewers that there are several limitations (some limitations due to lack of the data while some others are due to lack of techniques). This subsection will help the reader to judge the predictions of this model and at the same time it will help other researchers to make any advancement of the current study.

Author response:

We thank the reviewer for the suggestion, and we included a conclusion section with the limitations of the current work and some possible applications of the models once additional data becomes available. Moreover, to show the readers that the model is robust, consistent and reliable we added the FROG report, and published the model at the biomodels database (MODEL2408120001).

Conclusion (line 750)

In this work, diel multi-tissue models of *V. vinifera* were reconstructed by integrating tissue-specific omics data for leaf, stem and grapes. The iMS7199 model is available

in the BioModels database (https://www.ebi.ac.uk/biomodels/MODEL2408120001), together with the FROG (https://www.ebi.ac.uk/biomodels/curation/fbc) report that shows the model's robustness and reproducibility.

Likewise, the tissue-specific and diel models have also been published in the biomodels database (https://www.ebi.ac.uk/biomodels/MODEL2408160001). These models confirm the effect of different sulfate and nitrate concentrations on the metabolic content of the grapes. In addition, the flux distributions obtained from grape GSMMs were analysed by ML models and the most contributing reactions are involved in MEP and threonine biosynthesis and could represent putative biomarkers of the grape development stage.

The main limitation of these GSMMs is the lack of experimental data to define pseudo-reactions that represent the drain of macromolecules to create a new unit of biomass, and to validate the phenotype predictions. Nevertheless, these pseudo-reactions can be easily updated when actual data on the biomass composition from *V. vinifera* becomes available. In addition, the absence of significant differences between the green and mature grape models may be associated with the use of time-course omics data, which can hinder the identification of state-specific metabolic patterns. Hence, these models could obtain better results when applied to contrasting conditions. For instance, in future work, the model can be integrated with other datasets to investigate condition-specific metabolic phenotypes, such as responses to environmental stresses, like drought, or to understand disease mechanisms and developmentally regulated pathways, thereby offering a more comprehensive understanding of the plant's metabolism. Additionally, applying ML to analyse the fluxomics data predicted by the GSMMs can be greatly improved when larger omics datasets become available, allowing for more representative results.

MAIN MODEL:

Please forward the following instructions to the reviewers:

To access these models:

1. Please visit https://www.ebi.ac.uk/biomodels/login/auth
2. Log in with username reviewerForMODEL2408120001 and password JY0PJE
3. Access the model at this link: https://www.ebi.ac.uk/biomodels/MODEL2408120001
4. In case of problems, please email biomodels-net-support@lists.sf.net, indicating the username reviewerForMODEL2408120001.

CONTEXT SPECFIFIC MODELS:

Please forward the following instructions to the reviewers:

To access these models:

1. Please visit https://www.ebi.ac.uk/biomodels/login/auth
2. Log in with username reviewerForMODEL2408160001 and password UB57YM
3. Access the model at this link: https://www.ebi.ac.uk/biomodels/MODEL2408160001
4. In case of problems, please email biomodels-net-support@lists.sf.net, indicating the username reviewerForMODEL2408160001.

The authors may be requested (extending the queries of the third reviewer) to show how the traditional PCA like methods fail to reach the same conclusion what the authors have obtained using ML method.

Author response:

PCA is an unsupervised method that focuses on reducing the dimensionality of the data while capturing the variance and structure of the data without considering the target output. Hence, PCA may identify features that show high variance across all samples but are not necessarily useful for distinguishing the output classes. In addition, PCA linearly transforms the features which may not be ideal for non-linear data. On the other hand, the supervised methods used in this work focus on predicting the output class based on the input features, allowing the identification of the features that are most predictive of the output class. Therefore, we utilized t-SNE as it was more suitable for this task.
